# Development of an Oncogenic Driver Alteration Associated Immune-Related Prognostic Model for Stage I-II Lung Adenocarcinoma

**DOI:** 10.3389/fonc.2020.593022

**Published:** 2021-01-28

**Authors:** Jian-Zhao Xu, Chen Gong, Zheng-Fu Xie, Hua Zhao

**Affiliations:** Geriatrics Respiratory Medicine, The First Affiliated Hospital of Guangxi Medical University, Nanning, China

**Keywords:** tumor microenvironment, immune-related prognostic model, lung adenocarcinoma, oncogenic driver alterations, The Cancer Genome Atlas, Gene Expression Omnibus

## Abstract

Lung adenocarcinoma (LUAD) needs to be stratified for its heterogeneity. Oncogenic driver alterations such as *EGFR* mutation, *ALK* translocation, *ROS1* translocation, and *BRAF* mutation predict response to treatment for LUAD. Since oncogenic driver alterations may modulate immune response in tumor microenvironment that may influence prognosis in LUAD, the effects of *EGFR*, *ALK*, *ROS1*, and *BRAF* alterations on tumor microenvironment remain unclear. Immune-related prognostic model associated with oncogenic driver alterations is needed. In this study, we performed the Cox-proportional Hazards Analysis based on the L1-penalized (LASSO) Analysis to establish an immune-related prognostic model (IPM) in stage I-II LUAD patients, which was based on 3 immune-related genes (*PDE4B*, *RIPK2*, and *IFITM1*) significantly enriched in patients without *EGFR*, *ALK*, *ROS1*, and *BRAF* alterations in The Cancer Genome Atlas (TCGA) database. Then, patients were categorized into high-risk and low-risk groups individually according to the IPM defined risk score. The predicting ability of the IPM was validated in GSE31210 and GSE26939 downloaded from the Gene Expression Omnibus (GEO) database. High-risk was significantly associated with lower overall survival (OS) rates in 3 independent stage I-II LUAD cohorts (all *P* < 0.05). Moreover, the IPM defined risk independently predicted OS for patients in TCGA stage I-II LUAD cohort (*P* = 0.011). High-risk group had significantly higher proportions of macrophages M1 and activated mast cells but lower proportions of memory B cells, resting CD4 memory T cells and resting mast cells than low-risk group (all *P* < 0.05). In addition, the high-risk group had a significantly lower expression of *CTLA-4*, *PDCD1*, *HAVCR2*, and *TIGIT* than the low-risk group (all *P* < 0.05). In summary, we established a novel IPM that could provide new biomarkers for risk stratification of stage I-II LUAD patients.

## Introduction

Lung adenocarcinoma (LUAD) is the most common type of lung cancer that comprises around 40% of all lung cancer, and it is also one of the most aggressive and rapidly fatal tumor types (1). Molecular alterations play important roles in the genesis and development of lung cancer, especially in LUAD, for it often occurs in females and never-smokers, and LUAD can be classified according to the presence of specific mutually exclusive oncogene aberrations that drive carcinogenesis (2–5). Moreover, molecular alterations including *EGFR* mutation, *ALK* translocation, *ROS1* translocation, and *BRAF* mutation provide definite targets for drugs in precision medicine (6). However, similar to conventional chemotherapies, the development of resistance for these new-targeted drugs is still a major challenge for treatment effectiveness. Despite the success of targeted-based therapies, early diagnosis and surgical resection of early-stage disease remain the best opportunity for a cure, for that outcome varies differently between LUAD patients at early stage and advanced stage (7, 8). But in spite of its early stage of development, LUAD patients at stage I-II are at substantial risk for recurrence and death, even after complete surgical resection. Therefore, more indicators are urged to be evaluated for further stratified LUAD patients at stage I-II to provide precision treatment.

Tumor microenvironment (TME) plays an important role in cancers’ development include LUAD, which is constituted by varieties of immune and stromal cell types (endothelial cells, fibroblasts, etc.) and extracellular components they secrete (cytokines, growth factors, hormones, extracellular matrix, etc.), involved in cancer immunoediting, including elimination, equilibrium and escape (9–13). *EGFR* mutation has been demonstrated to correlate with an immunosuppressive TME in non-small-cell lung cancer (NSCLC), and *EGFR* tyrosine kinase inhibitors (TKIs) may modulate the immune response by regulating TME (14–20). Further, some studies showed immune checkpoint inhibitors have poor efficacy in NSCLC patients who harbor an *EGFR* mutation or *ALK* translocation, whereas they appear to be active in those with a *BRAF* mutation (21, 22). Therefore, we speculate that the poor response to treatment of LUAD patients harboring molecular alterations may be partly caused by the specific influences of these alterations on the composition of the TME, such as increase immunosuppressive cells or decreased immunoreactive cells. Thus, understanding the exact effects of molecular alterations on the cancer-associated immune microenvironment in LUAD is critical. In the current study, we downloaded gene expression data of stage I-II LUAD cohorts from The Cancer Genome Atlas (TCGA) database to study the relationship between *EGFR* mutation, *ALK* translocation, *ROS1* translocation, and *BRAF* mutation and immune TME in LUAD, and establish an immune-related prognostic model (IPM) for prognostic prediction in LUAD patients at stage I-II, which is a widely used method for diseases’ prognostic prediction in different type of solid tumors (23–26).

## Materials and Methods

### Data Sources

**Figure 1:**
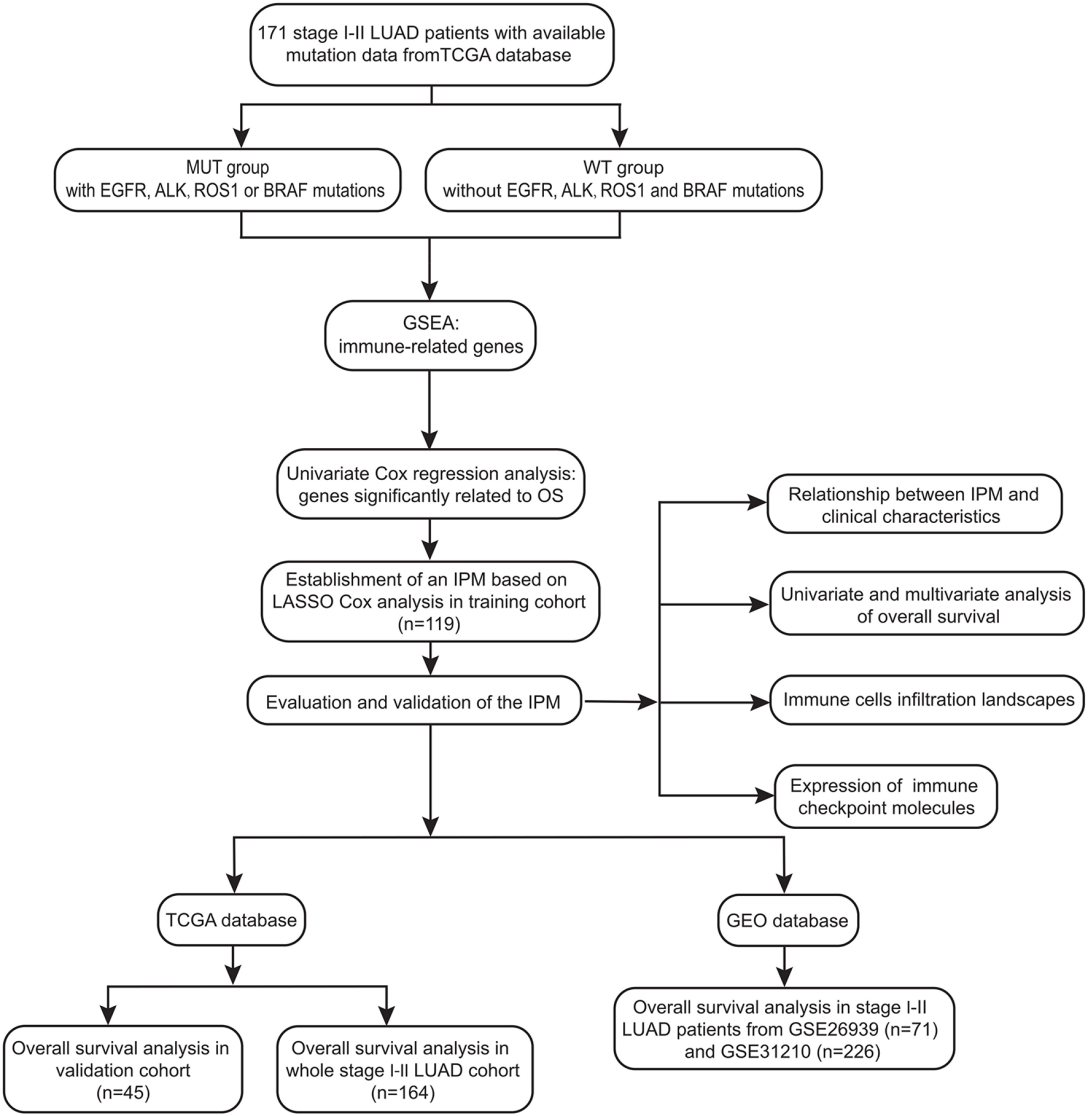
Workflow chart of data generation and analysis.

The study design and workflow were provided in **Figure 1**. The somatic mutation status (workflow type: VarScan2 Variant Aggregation and Masking), transcriptional profiles, and the corresponding clinical and overall survival (OS) data of 403 stage I-II LUAD patients were downloaded from The Cancer Genome Atlas (TCGA) database (https://portal.gdc.cancer.gov/). The gene expression profile was measured experimentally using the Illumina HiSeq 2000 RNA Sequencing platform. The gene symbols were annotated based on the Homo_sapiens. GRCh38.91.chr.gtf file (http://asia.ensembl.org/index.html). Log2 transformations were performed for all gene expression data. The study reported here in fully satisfies the TCGA publication requirements (http://cancergenome.nih.gov/publications/publicationguidelines).

For validation of the predict ability of the IPM established based on TCGA data, the gene expression profile-matrix files from GSE31210 based on platform GPL570 (Affymetrix Human Genome U133 Plus 2.0 Array) and GSE26939 based on platform GPL9053 (Agilent-UNC-custom-4X44K) and the individual clinical and OS information were downloaded from GEO database (https://www.ncbi.nlm.nih.gov/geo/). The obtained data were used according to the GEO data access policies. Both mRNA profile data and clinical feature data of stage I-II LUAD are made public. All analyses were carried out based on pertinent guidelines and regulations.

### Gene Set Enrichment Analysis (GSEA)

GSEA (Version:3.0; http://software.broadinstitute.org/gsea/index.jsp) was performed to identify different enriched immune-related genes between patients without *EGFR* mutation, *ALK* translocation, *ROS1* translocation, and *BRAF* mutation (WT) group and patients with *EGFR* mutation, *ALK* translocation, *ROS1* translocation, or *BRAF* mutation (MUT) group (27). An annotated gene set file (the expression signatures of the hallmark gene sets, each containing 50 specific gene sets derived by concentrating multiple gene sets from the Molecular Signatures Database (MSigDB) was selected as the reference gene set. The GSEA threshold for significantly enriched functional annotations was set at a *P* < 0.05, false discovery rate (FDR) < 0.25, and a normalized enrichment score > 1.5.

### Identification of Immune-Related Prognostic Signature

Univariate Cox regression analysis was performed using the R package “survival” to evaluate correlations between the expression levels of immune-related genes selected by GSEA and the OS of stage I-II LUAD patients in TCGA cohort. Immune-related genes with *P* < 0.05 by univariate Cox regression analysis were identified as alternative prognostic genes.

### Cox-Proportional Hazards Analysis Based on the L1-Penalized (LASSO) Analysis

LASSO with L1-penalty is a popular method for determining interpretable prediction rules that could be used to successfully address the collinearity problem (28). Therefore, a sub-selection among the immune-related genes that were significant in the univariate Cox regression analysis was determined by LASSO-penalized Cox regression analysis, which was performed by using the R package “glmnet” (Version: 2.0–16; https://cran.r-project.org/web/packages/glmnet/index.html), and genes were regarded as significant at *P* < 0.05. To be specific, stage I-II LUAD patients in TCGA cohort were randomly split into training and validation cohorts using a 7:3 ratio, then LASSO-penalized Cox regression analysis was performed in the training cohort, the tuning parameters were determined according to the expected generalization error estimated from 10-fold cross-validation and information-based criteria Akaike Information Criterion (AIC)/Bayesian Information Criterion (BIC), and the “min” lambda was adopted. Furthermore, the dataset was subsampled 1,000 times and genes that were repeated N ≥ 900 times were chosen. As a result, a relatively small number of immune-related genes involved in stage I-II LUAD patients’ prognosis with a weight of nonzero that was determined by shrinkage of the regression coefficient *via* the imposition of a penalty proportional to their size were identified.

### Establishment and Validation of an Immune-Related Prognostic Model (IPM)

Finally, an IPM based on key immune-related genes selected by LASSO Cox analysis was established in training cohort. By weighting the expression level of each immune gene (*RIPK2*, *PDE4B*, and *IFITM1*) to the regression coefficients of the multivariate Cox regression analysis, the risk score derived from the IPM was calculated by utilizing the “predict” function in R software to assess each patient’s risk level. The optimal cutoff of a risk score was identified by the maximum Youden’s index that was obtained from a receiver operating characteristic (ROC) curve, and patients with available survival data were separated into IPM defined high-risk and low-risk groups, respectively. The predictability of the IPM was evaluated by area under ROC (AUC), which calculated the proportions of concordant pairs among all pairs of observations with 1.0 indicating perfect prediction accuracy. The higher the value of the AUC is, the better the predictability of the model. In addition, the performance of the IPM in predicting prognosis was validated by survival analysis in 2 independent stage I-II LUAD cohorts from GEO database.

### Estimation of Immune Cell Type Fractions

The Cell type Identification by Estimating Relative Subsets of RNA Transcripts (CIBERSORT) is used for characterizing the cell composition of complex tissues based on their gene expression profiles, and it is highly consistent with ground-truth estimations in many cancers (29). A leukocyte-gene signature matrix consisting of 547 genes and termed LM22 was used to distinguish 22 immune cell types; these types contained myeloid subsets, natural killer (NK) cells, plasma cells, naive and memory B cells, and seven T cell types. We uploaded normalized gene expression data with standard annotation files to the CIBERSORT web portal and utilized CIBERSORT in combination with the LM22 signature matrix to estimate the fractions of 22 human hematopoietic cell phenotypes between stage I-II LUAD samples in high-risk group and in low-risk group. The sum of all estimated immune cell type fractions was equal to 1 for each sample. The threshold was set at *P* < 0.05 and the final CIBERSORT output was subsequently analyzed.

### Statistical Analysis

Comparisons of immune cell type fractions and checkpoints, including *CTLA-4*, *PDCD1* (also known as *PD-1*), *CD274* (also known as *PD-L1*), *HAVCR2* (also known as *TIM3*), *LAG3*, and *TIGIT* between high-risk and low-risk groups, was performed using the Mann-Whitney U test. Pairwise comparisons of the variables between groups were performed using the Mann-Whitney U test for continuous variables and the Fisher’s exact test for categorical variables. The log-rank test and Kaplan-Meier survival analysis were used to test the predictive ability of the IPM in training cohort, validation cohort, and the whole stage I-II LUAD cohort. Additionally, we conducted univariate Cox regression, and variables associated with *P* ≤ 0.20 in the univariate analysis were entered into a multivariable Cox regression analysis to check whether the IPM was an independent prognostic factor within the available data. The level for a statistically significant difference was set at *P* < 0.05. The SPSS 21.0 software package (SPSS Inc.) and GraphPad Prism 5 (GraphPad Software Inc.) were used for data analysis.

## Results

### *EGFR*, *ALK*, *ROS1*, and *BAFR* Mutations Associated Immune Profile in Stage I-II LUAD Patients in the TCGA Cohort

**Figure 2:**
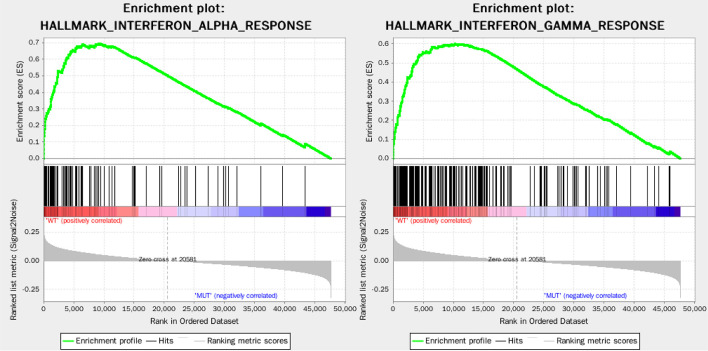
Enrichment plots of two immune-related gene sets that were significantly enriched in the WT group.

Among 403 stages I-II LUAD patients in TCGA database, a total of 171 patients with available gene expression profile and mutation status were analyzed. Their *EGFR*, *ALK*, *ROS1*, and *BRAF* mutation frequencies were 14.6% (25/171), 8.8% (15/171), 5.8% (10/171), and 8.8% (15/171), respectively. A total of 110 patients had no mutation in *EGFR*, *ALK*, *ROS1*, and *BRAF* mutation and were categorized as WT group (n=110), and the remaining 61 patients had at least one somatic mutation (*EGFR*, *ALK*, *ROS1*, or *BRAF*) and were categorized as MUT group (n=61). GSEA analysis of WT group and MUT group showed the immune response of WT group was markedly stronger than MUT group, that 50 gene sets were upregulated in lung adenocarcinoma, and 30 gene sets were upregulated in WT group, among which 2 immune-related gene sets were greatly enriched, with normalized *P* < 0.05 (**Figure 2**). Thus, these two top-ranking functions, namely, INTERFERON_ALPHA_RESPONSE (normalized enrichment score: NES = 1.82, size = 95, *P* = 0.023) and INTERFERON_GAMMA_RESPONSE (NES = 1.79, size = 198, *P* = 0.049) were selected, and 220 non-repetitive immune-related genes were obtained from these two immune-related processes.

### Identification of Immune-Related Genes Associated With Survival

Of 171 stage I-II LUAD patients in TCGA database, 164 patients had survival information and were performed survival analysis. The general information of 164 patients was summarized in **Table 1**. Their median follow-up period was 800 days (range: 14–7248 days) and the 3-year OS rate was 69.9% (95% confidence interval [CI]: 60.5–77.5%).

**Table 1:** Univariate Cox regression analysis results of seven prognostic immune-related genes significantly associated with overall survival in stage I-II LUAD patients in TCGA cohort.

Gene	Hazard ratio	P value
PDE4B	0.66	0.010
RIPK2	1.64	0.012
HLA.DMA	0.76	0.018
CIITA	0.75	0.020
ITGB7	0.71	0.024
IFITM1	0.82	0.030
CD74	0.82	0.036

The results of Univariate Cox regression analysis in 164 patients revealed that 7 of the 220 immune-related genes were significantly related to OS (**Table 1**). Then 164 patients were randomly split into training and validation cohorts using a ratio of 7:3, corresponding to 119 and 45 total patients, respectively. Further, LASSO-penalized Cox regression analysis was performed in training cohort and only 3 of 7 immune-related genes showed significant prognostic signatures (i.e., *P* < 0.05), that is Phosphodiesterase 4B (*PDE4B*), Receptor-interacting protein kinase 2 (*RIPK2*), and Interferon-inducible transmembrane protein 1 (*IFITM1*).

### Establishment and Evaluation of an IPM to Predict Patient Outcomes in the Training Cohort of TCGA Database

**Figure 3:**
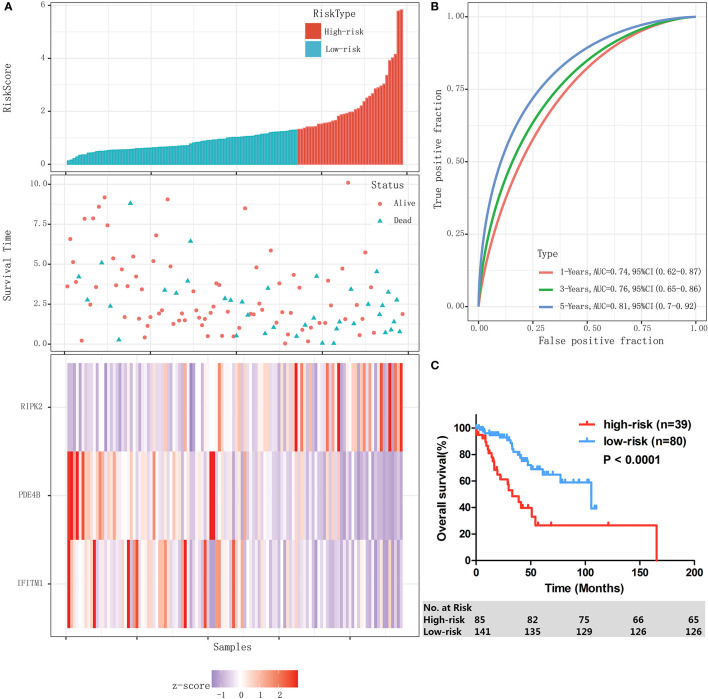
Establishment and evaluation of an IPM in the training cohort. **(A)** Risk score distribution and gene expression data. **(B)** Time-dependent ROC curve of the IPM for training cohort. **(C)** Kaplan-Meier survival of the IPM for training cohort.

Then an IPM was established based on 3 immune-related genes. By weighting the expression level of each immune gene to the regression coefficients of the multivariate Cox regression analysis, a risk score system was established to predict patient survival by using the “predict” function in R software in training cohort (n=119). The risk score was calculated for each patient. The risk score distribution and gene expression data were shown in **Figure 3A**. The IPM achieved an AUC of 0.74 at 1 year, 0.76 at 3 years, and 0.81 at 5 years (**Figure 3B**).

The ROC curves showed that 1.29 had a maximum Youden’s index (0.31) among all values. Thus 1.29 was identified as the optimal cutoff value of the risk score, risk score ≥ 1.29 and < 1.29 were defined as high-risk group and low-risk group, respectively. In the entire cohort, 39 (32.8%) and 80 (67.2%) patients were individually categorized as high-risk and low-risk group. As a result, patients in high-risk group had significantly lower 3-year OS rate than the patients in low-risk group (48.6% [95% CI: 29.4–65.3%] vs 84.2% [95% CI: 71.3–91.6%], *P* < 0.0001, **Figure 3C**).

### Authentication of the IPM in Validation-Cohort and the Whole Stage I-II LUAD Cohort of TCGA Database

**Figure 4:**
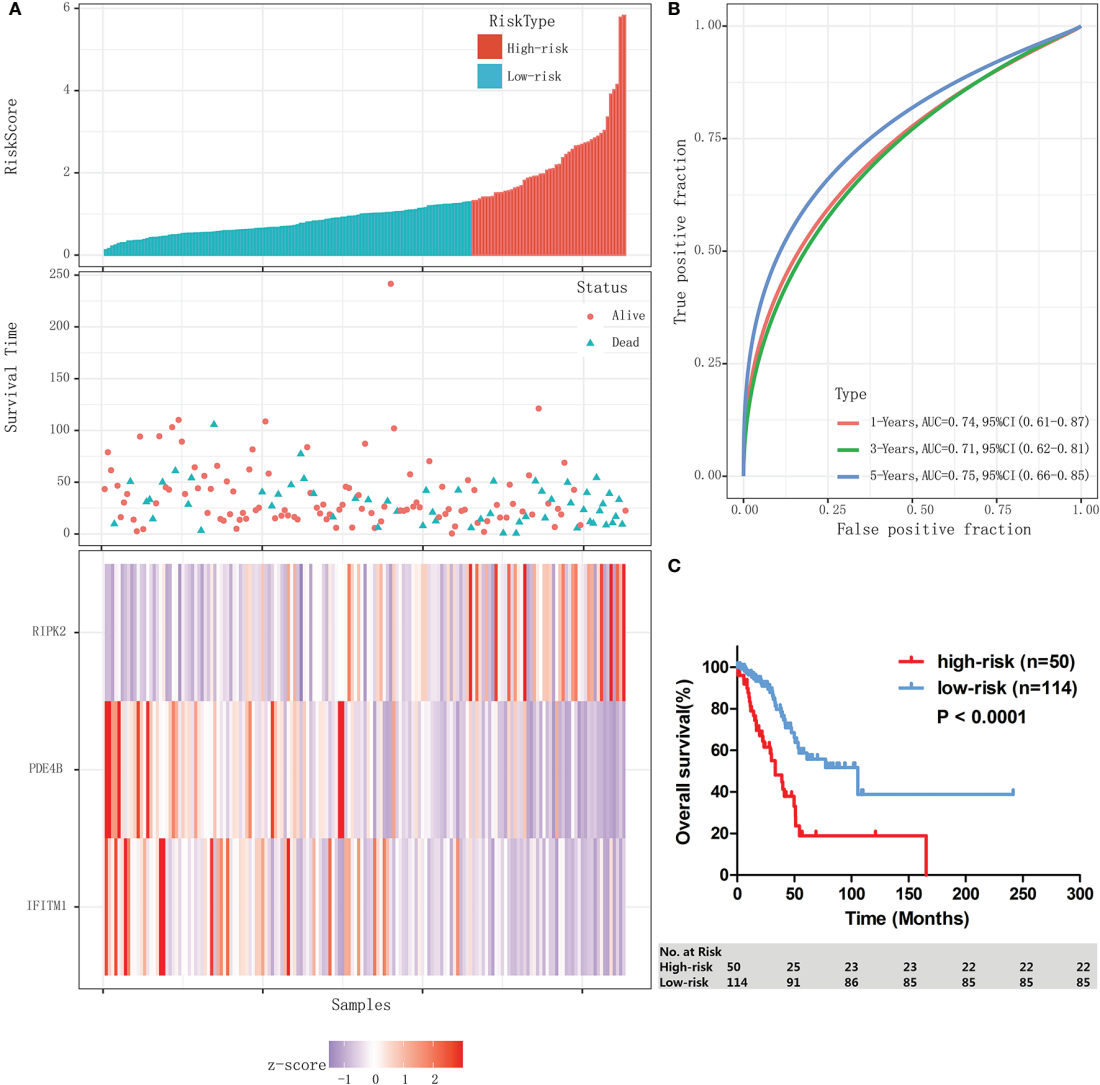
Validation of the IPM in the whole stage I-II LUAD cohort. **(A)** Risk score distribution and gene expression data. **(B)** Time-dependent ROC curve of the IPM for the whole stage I-II LUAD cohort. **(C)** Kaplan-Meier survival of the IPM for the whole stage I-II LUAD cohort.

The performance of the IPM was assessed in validation cohort (n=45) and the whole stage I-II LUAD cohort (n=164) individually. With the same formula and the same cutoff obtained from training cohort (n=119), patients in validation cohort and the whole stage I-II LUAD cohort were individually divided into high-risk group and low-risk group. In validation cohort, patients assigned to high-risk group (n=11) had a significantly lower 3-year OS rate than those assigned to low-risk group (n=34) (46.8% [95% CI: 13.5–74.9%] vs 74.1% [95% CI: 49.7–87.9%], *P* = 0.020). Similar results were found in the whole stage I-II LUAD cohort; the risk score distribution and gene expression data of the whole stage I-II LUAD cohort are shown in **Figure 4A**, and the IPM achieved an AUC of 0.74 at 1 year, 0.71 at 3 years, and 0.75 at 5 years (**Figure 4B**). High-risk patients (n=50) had significantly shorter 3-OS rate than low-risk patients (n=114) (48.2% [95% CI: 31.3–63.2%] vs 81.3% [95% CI: 70.5–88.5%], *P* < 0.0001, **Figure 4C**).

### Relationship Between IPM-Defined Risk and Patient Characteristics in the TCGA Cohort

As shown in **Table 2**, of 164 stage I-II LUAD patients in the TCGA cohort, IPM-high risk (IPM-HR) was significantly related to advanced age (*P* < 0.0001), survival status (*P* < 0.0001), and N stage (*P* < 0.0001). However, the IPM defined risk had no relationship with sex, smokers or never smokers, T stage, residual tumor, *EGFR* mutation, *ALK* mutation, *ROS1* mutation, *BRAF* mutation, and WT/MUT groups (all *P* > 0.05).

**Table 2:** Relationship between IPM-defined risk and patient characteristics at diagnosis in Stage I-II LUAD patients in the TCGA cohort.

Variable	All	IPM defined risk		P value
		IPM-LR	IPM-HR	
Number of patients	164	114	50	
Age (year, median, range)	67 (41–86)	68 (41–86)	65.5 (42–85)	<0.0001
Males (%)	69 (42.1%)	46 (40.4%)	23 (46%)	0.61
Smoker, n (%)				
Smokers	133 (81.1%)	93 (81.6%)	40 (80.0%)	0.63
Never smokers	25 (15.2%)	16 (14.0%)	9 (18.0%)	
Not Available	6 (3.7%)	5 (4.4%)	1 (2.0%)	
Survival status, n (%)				
alive	106 (64.6%)	85 (74.6%)	21 (42%)	<0.0001
dead	58 (35.4%)	29 (25.4%)	29 (58%)	
T stage, n (%)				
T1	58 (35.4%)	45 (39.5%)	13 (26%)	0.11
T2-3	106 (64.6%)	69 (60.5%)	37 (74%)	
N stage, n (%)				
N0	124 (75.6%)	89 (78.1%)	11 (55.0%)	<0.0001
N1	35 (21.3%)	20 (17.5%)	15 (35.0%)
Nx	5 (3%)	5 (4.4)	0 (0%)	
Residual tumor, n (%)				
R0	116 (70.7%)	83 (72.8%)	33 (66.0%)	0.84
R1	5 (3.0%)	3 (2.6%)	2 (4.0%)
Rx	3 (1.8%)	2 (1.8%)	1 (2.0%)	
Not Available	40 (24.4%)	26 (22.8%)	14 (28.0%)	
*EGFR*, n (%)				
Mutation	25 (15.2%)	16 (14.0%)	9 (18.0%)	0.64
Wild type	139 (84.8%)	98 (86.0%)	41 (82.0%)
*ALK*, n (%)				
Mutation	15 (9.1%)	10 (8.8%)	5 (10.0%)	0.78
Wild type	149 (90.9%)	104 (91.2%)	45 (90.0%)
*ROS1*, n (%)				
Mutation	10 (6.1%)	7 (6.1%)	3 (6.0%)	0.97
Wild type	154 (93.1%)	107 (93.9%)	47 (94.0%)
*BRAF*, n (%)				
Mutation	15 (9.1%)	10 (8.8%)	5 (10.0%)	0.80
Wild type	149 (90.9%)	104 (91.2%)	45 (90.0%)
WT/MUT Group, n (%)				
MUT group	58 (35.4%)	38 (33.3%)	20 (40.0%)	0.41
WT group	106 (64.6%)	76 (66.7%)	30 (60.0%)

### The IPM Independently Predicted Poor Outcomes for Stage I-II LUAD Patients in TCGA Dataset

As shown in **Table 3**, univariate analysis was performed in the whole stage I-II LUAD cohort. In addition to the IPM, N classification, UICC stage, age, neoplasm cancer status, and residual tumor status were all effective for predicting the OS rate of stage I-II LUAD patients (all *P* < 0.05). Multivariable analysis showed that high-risk score of the IPM and a positive tumor finding during the follow-up visit were all independent adverse prognostic factors for OS in stage I-II LUAD patients (**Table 4**).

**Table 3:** Univariate analysis of OS in stage I-II LUAD patients in TCGA.

Variable	OS	
	HR(95%CI)	P value
High-risk	3.08(1.83–5.18)	<0.0001
N_1_	3.09(1.80–5.30)	<0.0001
Stage II	2.94(1.72–5.01)	<0.0001
Age > 70y	1.71(1.01–2.89)	0.045
With tumor	8.47(4.47–16.02)	<0.0001
Residual tumor	4.62(1.59–13.39)	0.005

**Table 4:** Independent prognostic factors for OS in stage I-II LUAD patients in TCGA.

	No. of patients	HR(95%CI)	P value
IPM			
High-risk	50	2.63(1.24–5.56)	0.011
Low-risk	114	1.00	
Neoplasm cancer status			
With tumor	27	6.13(2.93–12.84)	<0.0001
Tumor free	104	1.00	

### Validation of the IPM in GEO Stage I-II LUAD Cohorts by Kaplan–Meier Curve Analysis

**Figure 5:**
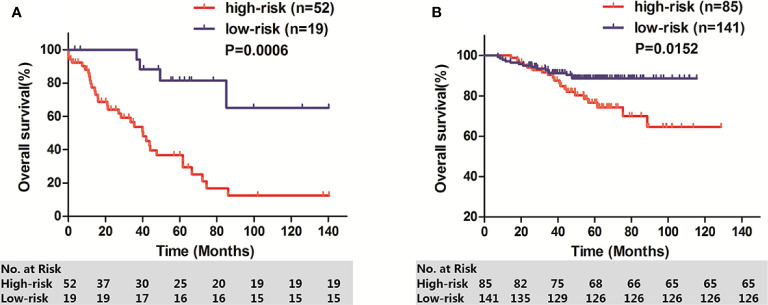
OS of two GEO stage I-II LUAD cohorts. **(A)** GSE26939; **(B)** GSE31210.

To determine whether the IPM was robust, 71 stage I-II LUAD patients from GSE26939 and 226 stage I-II LUAD patients from GSE31210 were used to verify the prognostic significance of IPM defined risk. Patients in each cohort were calculated IPM defined risk score and divided into high-risk group and low-risk group based on the individual ROC curve determined cutoff value. Consistent with the results in TCGA cohort, patients in high-risk group had significantly lower OS rate than those in low-risk group, respectively (GSE26939: *P* = 0.0006, **Figure 5A**; GSE31210: *P* = 0.0152, **Figure 5B**).

### The Immune Landscape Between the High- and Low-Risk Group of Stage I-II LUAD Patients

**Figure 6:**
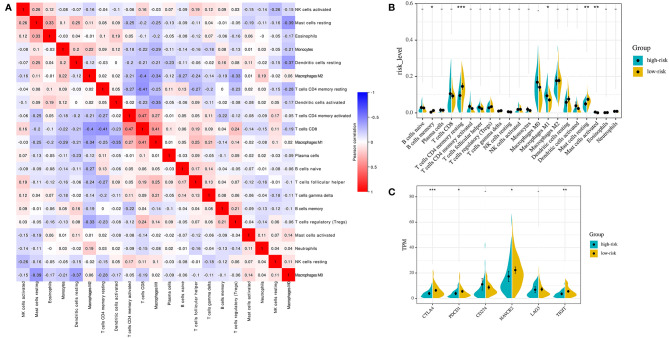
Immune landscapes of the IPM defined risk **(A)** Correlation matrix of all 22 immune cell proportions. **(B)** Violin plots visualizing significantly different immune cells between high-risk and low-risk patients. **(C)** Violin plots visualizing significantly different immune checkpoints between high-risk and low-risk patients. *P < 0.05,** P < 0.01, ***P < 0.001.

For the whole 164 stage I-II LUAD patients in TCGA cohort, the proportions of 22 immune cell types were estimated between high-risk and low-risk groups using the CIBERSORT method. The proportions of different subpopulations of tumor-infiltrating immune cells were weakly to moderately correlated (**Figure 6A**). High-risk group patients had significantly higher proportions of macrophages M1 (*P* = 0.024) and activated mast cells (*P* = 0.009), and lower proportions of memory B cells (*P* = 0.038), resting CD4 memory T-cells (*P* = 0.001), and resting mast cells (*P* = 0.001) than the low-risk group patients (**Figure 6B**).

Then, we investigated the expression of critical immune checkpoint molecules (*CTLA-4*, *PDCD1*, *CD274*, *HAVCR2*, *LAG3*, and *TIGIT*) between high-risk group and low-risk group patients. The expression of *CTLA-4*, *PDCD1*, *HAVCR2*, and *TIGIT* in high-risk group was significantly lower than that in low-risk group in the whole 164 stage I-II LUAD patients in TCGA cohort (*CTLA-4*: *P* = 0.001; *PDCD1*: *P* = 0.043; *HAVCR2*: *P* = 0.030; *TIGIT*: *P* = 0.021, **Figure 6C**).

## Discussion

In the current study, we developed an IPM associated with oncogenic driver alterations frequently occur in LUAD based on TCGA database, and demonstrated that the IPM defined risk independently predicted overall survival in stage I-II LUAD, which validated by GSE databases.

For the first time, we identification immune relevant genes significantly enriched in stage I-II LUAD patients without *EGFR* mutation, *ALK* translocation, *ROS1* translocation, and *BRAF* mutation to establish an IPM. Though the prognostic impact of *EGFR* mutation, *ALK* translocation, *ROS1* translocation, and *BRAF* mutations remains controversial in LUAD, targeted therapy remains the best strategy to treat lung cancer patients who harbor these oncogenic driver alterations, which were specifically included in NCCN guideline (5, 30). Thus, only *EGFR* mutation, *ALK* translocation, *ROS1* translocation, and *BRAF* mutation status were considered to be included for identifying candidate genes to develop IPM in the current study. After performing univariate Cox regression and LASSO Cox analysis of immune relevant genes significantly enriched in WT group in according to GSEA analysis, we established an IPM composed of 3 genes expression with individual weight. Then the optimal cutoff value obtained from ROC curve analysis was used to divide patients into high-risk and low-risk groups in each cohort, respectively. The survival analysis demonstrated that IPM defined high-risk was an independent poor prognostic factor in stage I-II LUAD patients in TCGA cohort. Since our IPM is based on tumor microenvironment-related genes, although its establishment is related to *EGFR* mutation, *ALK* translocation, *ROS1* translocation, and *BRAF* mutation, it is applicable to all patients with or without these mutations in stage I-II LUAD. Furthermore, this impact was individually validated in 2 independent cohorts of stage I-II LUAD patients downloaded from GEO databases.

The IPM was consisted with *RIPK2*, *PDE4B*, and *IFITM1* gene. *RIPK2* gene encodes a member of the receptor-interacting protein (RIP) family of serine/threonine protein kinases, which contains a C-terminal caspase activation and recruitment domain (CARD) and is a component of signaling complexes in both the innate and adaptive immune pathways (31). It is a central adaptor kinase in the NOD pathway, potent activator of NF-kappaB, and inducer of apoptosis in response to various stimuli, and it also has novel roles in cancer cell migration and invasion (32–34). Several studies have revealed the potential of RIPK inhibitors in amelioration of inflammatory signalling and/or inflammatory cell death, and the *RIPK2* inhibitors can potently inhibited the proliferation of cancer cells (32, 34), but there are few studies that have focused on the prognostic impact of *RIPK2* on lung cancer. *PDE4B* gene is a member of the type IV, cyclic AMP (cAMP)-specific, cyclic nucleotide phosphodiesterase (PDE) family, and the encoded protein of *PDE4B* regulates the cellular concentrations of cyclic nucleotides and thereby play a role in signal transduction. *PDE4B* is abundant in leukocytes, a series of studies have reported an important role for cAMP and *PDE4B* in TNF-α expression after LPS stimulation, neutrophil recruitment, and apoptosis or T cell function (35–39). Inflammatory stimuli can also enhance *PDE4B* activity through their elevation of the transcription of *PDE4B* mRNA and increased *PDE4B* (40, 41). The expression of *PDE4B* varied among different cancer type and its prognostic significance remains controversial in cancer (42). *PDE4B* expression was found to be increased in non-small cell lung cancer tissues (43), and additional study highlights *in vitro* findings that specific *PDE4B* inhibition is cytotoxic in lung cancer cells (44). *IFITM1* encodes a protein that is a member of the interferon-inducible transmembrane protein family and was initially known as a leukocyte antigen, a part of the membrane complex involved in the transduction of antiproliferative and homotypic adhesion signals in lymphocytes (45–47). *IFITM1* plays an important role in the progression of cancer, including that it promotes tumor cell proliferation, invasion, metastasis, angiogenesis, and therapeutic resistance, including endocrine therapy, chemotherapy, and radiotherapy resistance, and it was always served as poor prognostic biomarkers for many cancers (48). Several studies showed that *IFITM1* critically regulates epidermal growth factor receptor-mediated signaling in non-small cell lung cancer models and is associated with a poor prognosis of patients with adenocarcinoma (49–51). However, some studies have shown that *IFITM1* expression has a rather beneficial prognosis (52). In addition, the *IFITM1* can inhibit virus infections by preventing virus membrane fusion with cells and by inhibiting fusion of infected cells (syncytialization) (53, 54). The expression of *RIPK2* was poor prognostic marker, whereas the expressions of *PDE4B* and *IFITM1* seemed favorable prognostic factors in our developed and validated IPM, and none of them has ever been systematically evaluated in stage I-II LUAD to date. Considering the published studies, we suspected that these 3 genes may have crucial function in modulating the immune response of TME on LUAD, which may depend on the context of the cancer, could be regarded as individual targets. Moreover, these 3 genes may provide better performance in combination, depending on their immune properties and prognostic significance in stage I-II LUAD.

Immunosuppressive networks are established to evade antitumor immune response during tumor development in immune-competent hosts by selecting less immunogenic cancer cells (55, 56). Disorder of the immunoreactive cells such as T cells follicular helper and immunosuppressive molecules and cells such as Treg cells and tumor-associated macrophages, as well as decreasing the expression of cancer antigens, are immunosuppressive mechanisms of cancers (57, 58). In the current study, we found that high-risk group patients generally had higher fractions of macrophages M1 and activated mast cells, and significantly lower proportions of memory B cells, resting CD4 memory T cells, and resting mast cells compared to the low-risk patients. The results indicate that patients in the high-risk group and low-risk group may have different mechanisms of tumor immune response. It has been confirmed that T cells CD4 memory resting can be further differentiated and confer various functions, including blocking CD8+T cell activation and NK cell killing, suppressing harmful immunological reactions to self-antigens and foreign antigens, and aiding CD8+T cells in tumor rejection (59, 60). The above results suggest that the poorer prognosis for high-risk LUAD patients may be due to higher immunosuppression and lower immunoreactivity in TME, thus promoting the development of tumor. It needs to be further confirmed by immune-profiling by flow cytometry in tumor tissue to be of relevant value in the future research.

Immune checkpoint molecules have been demonstrated play important roles in anti-tumor T-cell activity (61–63). Immunotherapy can stimulate cell-mediated immunity to recognize and destroy cancer cells by modulating T-cell function and targeting relevant mechanisms of immune resistance, such as immune inhibitory molecules in the tumor microenvironment (64). Both inhibitory of checkpoints (*CTLA-4* and *PDCD1*) commonly seen on activated T-cells have been found to be the most reliable targets for the treatment of cancer (65–67). In addition to the expression of *CTLA-4* and *PDCD1*, tumor-infiltrating lymphocytes (TILs) express a number of other co-inhibitory receptors, including *HAVCR2* and *TIGIT* (68, 69), providing additional targets that could be exploited for inducing anti-tumor immune responses. In the current study, we found that the low-risk stage I-II LUAD patients had significantly higher expression of *CTLA-4*, *PDCD1*, *HAVCR2*, and *TIGIT* than the high-risk patients. Since biomarkers can be used to identify patients who are more likely to respond to single-agent immune checkpoint inhibitors (70). Thus, the IPM can distinguish patients with a different expression of immune checkpoints, and may provide a novel immunotherapeutic strategy for stage I-II LUAD patients after more prospective studies are completed. In addition, it also indicates that the tumor immune microenvironment may be involved in the prognosis of stage I-II LUAD.

Overall, we established and validated an IPM based on 3 immune related genes associated with *EGFR* mutation, *ALK* translocation, *ROS1* translocation, and *BRAF* mutation status, which independently predicted the overall survival of stage I-II LUAD patients. High-risk was related to lower proportion of resting CD4 memory T cells and lower expression of checkpoint molecules *CTLA-4*, *PDCD1*, *HAVCR2*, and *TIGIT*. The current IPM may provide a new biomarker for stratification and immunotherapeutic strategies of stage I-II LUAD. To further validate the IPM, prospective studies with larger sample sizes, more detailed treatment information are warranted, and further studies on the mechanism of IPM are needed.

## Data Availability Statement

Publicly available datasets were analyzed in this study. These can be found in The Cancer Genome Atlas (https://portal.gdc.cancer.gov/) and GEO databases (https://www.ncbi.nlm.nih.gov/geo/).

## Author Contributions

CG designed the research. Material preparation, data collection, and analysis were performed by J-ZX, Z-FX, and HZ. The first draft of the manuscript was written by J-ZX. CG revised the manuscript. All authors contributed to the article and approved the submitted version.

## Funding

This study was supported by grant from the Guangxi health committee self-raised Fund of China (Z20190800).

## Conflict of Interest

The authors declare that the research was conducted in the absence of any commercial or financial relationships that could be construed as a potential conflict of interest.
